# Kidney micro-organoids in suspension culture as a scalable source of human pluripotent stem cell-derived kidney cells

**DOI:** 10.1242/dev.172361

**Published:** 2019-03-07

**Authors:** Santhosh V. Kumar, Pei X. Er, Kynan T. Lawlor, Ali Motazedian, Michelle Scurr, Irene Ghobrial, Alexander N. Combes, Luke Zappia, Alicia Oshlack, Edouard G. Stanley, Melissa H. Little

**Affiliations:** 1 Murdoch Children's Research Institute, Flemington Rd, Parkville, Victoria 3052, Australia; 2 Department of Paediatrics, The University of Melbourne, Parkville, Victoria 3010, Australia; 3 Department of Anatomy and Neuroscience, The University of Melbourne, Parkville, Victoria 3010, Australia; 4 School of Biosciences, Faculty of Science, The University of Melbourne, Parkville, Victoria 3010, Australia

**Keywords:** Kidney, Nephron, Organoid, Pluripotent stem cell, Single cell profiling, Suspension culture, Kidney micro-organoid

## Abstract

Kidney organoids have potential uses in disease modelling, drug screening and regenerative medicine. However, novel cost-effective techniques are needed to enable scaled-up production of kidney cell types *in vitro*. We describe here a modified suspension culture method for the generation of kidney micro-organoids from human pluripotent stem cells. Optimisation of differentiation conditions allowed the formation of micro-organoids, each containing six to ten nephrons that were surrounded by endothelial and stromal populations. Single cell transcriptional profiling confirmed the presence and transcriptional equivalence of all anticipated renal cell types consistent with a previous organoid culture method. This suspension culture micro-organoid methodology resulted in a three- to fourfold increase in final cell yield compared with static culture, thereby representing an economical approach to the production of kidney cells for various biological applications.

## Abstract (precis)

**Summary:** A modified culture format offers a simple and cost-effective method for expansion of hPSC-derived kidney cells, facilitating scale-up of kidney cell types *in vitro* for biomedical applications.

## INTRODUCTION

The directed differentiation of human pluripotent stem cells (hPSCs), including both induced pluripotent stem cells (iPSCs) and embryonic stem cells (hESCs), to distinct cellular endpoints has enabled the generation of complex organoid models for a variety of human tissues, including the kidney. Several protocols have been described to generate kidney tissue from hPSCs (Taguchi et al., 2014; Morizane et al., 2015; Takasato et al., 2015; Freedman et al., 2015; Taguchi and Nishinakamura, 2017), with variations in basal growth media, growth factor combinations and concentration (including the use of BMP4, FGF2, FGF9, BMP7, activin A), protocol duration (12 to 25 days) and culture format (Morizane and Bonventre, 2017). As for other hPSC directed differentiation protocols, the resulting component cell types represent a foetal stage of differentiation, for which transcriptional profiling suggests equivalence to trimester 1 human kidney development (Takasato et al., 2015). Although such protocols represent a source of human kidney cell types for disease modelling, drug screening, cellular therapy or tissue bioengineering, these protocols generate low numbers of final cells at a relatively high cost and have not been optimised for scale-up to the extent that will be required for potential cellular therapy or high-content screening approaches.

We have previously defined a stepwise induction protocol that directs differentiation of hPSCs through posterior primitive streak to intermediate mesoderm (IM), the tissue of origin for the kidney (Takasato et al., 2015, 2016). After dissociation and re-aggregation for micromass culture at an air-media interface, the resulting cultures begin to form early nephrons within a surrounding stroma that contains a vascular plexus and pericytic populations. The resulting kidney organoids contain at least nine distinct cell types. Organoids have been cultured on Transwell filters (up to nine organoids per Transwell) for up to 18 days after initial patterning in a monolayer format for 7 days. Commencing as a micromass of 5×10^5^ cells aggregated at day 7, each organoid reaches 3-5 mm in diameter and up to ∼200 µm in height at day 7+18 of the protocol (Takasato et al., 2016), representing a suboptimal culture format for oxygen diffusion. Indeed, prolonged culture in this format (until day 7+53) does not result in improved maturation of the component cell types (van den Berg et al., 2018) but leads to an apparent reduction in the ratio of epithelial to stromal cell types, reducing the yield of kidney epithelium per unit mass. As a result, improvements in culture methods are needed to facilitate scale-up of kidney cells types *in vitro* for biomedical applications.

We describe here a simple and cost effective method for the generation of large numbers of kidney micro-organoids via a suspension culture approach. In contrast to our previous method, cellular aggregates are formed at the IM stage of differentiation (day 7) as a result of dissociation and low speed swirling of monolayers before culture in low adhesion culture plates. This results in the formation of 8000-10,000 kidney micro-organoids. After 18 days in suspension culture, each micro-organoid comprises six to ten nephrons with evidence of early patterning and segmentation, including the formation of proximal and distal epithelium and glomeruli that contain podocytes. Importantly, single cell transcriptional profiling revealed equivalence between micro-organoids and standard organoids with respect to cellular diversity and maturity. Using this approach for directed differentiation resulted in a cell expansion of 30- to 40-fold across 21 days of culture, representing a three- to fourfold improvement in yield and a 75% reduction in cost per million organoid-derived kidney cells compared with our previous approach.

## RESULTS

### Generation of kidney micro-organoids

**Fig. 1.**
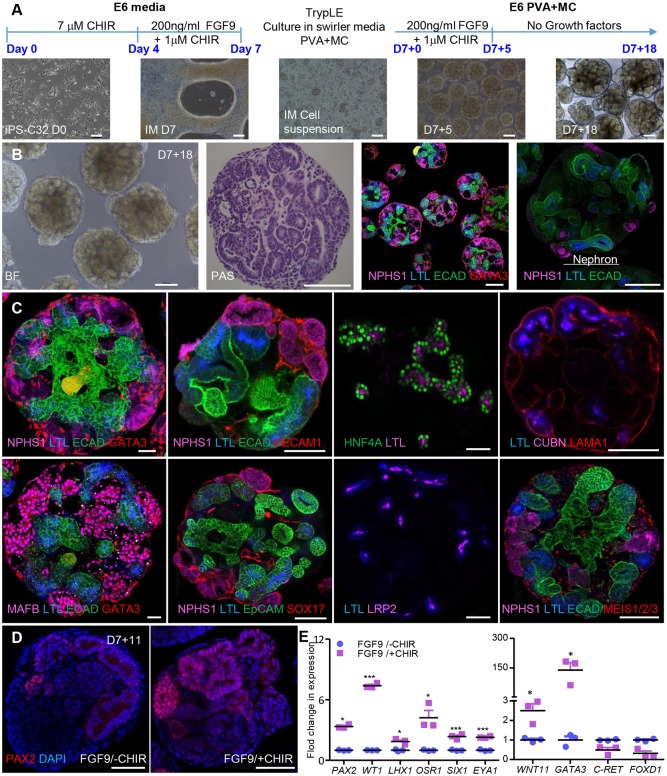
**Generation of kidney micro-organoids in suspension culture.** (A) Outline of the kidney micro-organoid differentiation protocol with images from a differentiation performed using CRL1502.C32 cells. (B) Bright-field image and PAS staining of kidney micro-organoids in suspension on day 7+18 (left), and overview confocal immunofluorescence image showing the different nephron segments in multiple organoids and magnified confocal image showing the entire nephron structure within an organoid (right). (C) Confocal immunofluorescence images of nephron compartments within day 7+18 kidney micro-organoids; podocytes (NPHS1+ and MAFB+), proximal tubules (LTL+, CUBN+, LRP2+ and HNF4A+), distal tubules (ECAD), collecting duct (ECAD+ GATA3+) and endothelial cells (SOX17+ and PECAM1+) (scale 50 µm). (D) Confocal immunofluorescence for PAX2 for ±1 µM CHIR99021 treatment (scale 50 µm). (E) Bar graphs showing average fold change for IM gene expression profiling by qPCR on day 7+0 for ±1 µM CHIR99021. Data are mean±s.e.m. *P<0.05, ***P<0.001; determined using two-tailed unpaired *t*-test. Scale bars: 50 µm (A); 100 µm (B); 50 µm (C,D).

Large-scale production of hPSC-derived kidney cell types from organoid cultures will require a quality controlled and cost-effective production approach. In order to address these issues, we modified our previous protocol for generating standard kidney organoids (Takasato et al., 2015, 2016) to develop a simple and effective protocol for the generation of large numbers of kidney micro-organoids from hPSCs, including both iPSC and hESC lines (Fig. 1A; Fig. S1A). Briefly, IM was derived by activating canonical Wnt signalling using the GSK3β inhibitor CHIR99021, followed by the addition of 200 ng/ml FGF9/heparin in Matrigel-coated six-well plate monolayer cultures, as previously described (Takasato et al., 2016). At day 7, the monolayer cultures of IM cells were exposed to EDTA or TrypLE Select and the resulting cell suspension was subjected to low speed (60 rpm) swirling on an orbital shaker in the presence of differentiation media (basal media that contains FGF9+heparin±CHIR99021) with 0.1% polyvinyl alcohol (PVA) and methyl cellulose (MC) to form cell aggregates using low adhesion 6 cm^2^ cell culture dishes. Within 24 h, kidney micro-organoids of 20-40 µm diameter formed. Kidney micro-organoids were subsequently cultured in the same medium until day 7+5, after which FGF9 and CHIR99021 were removed. After 18 days post-aggregation (day 7+18), each kidney micro-organoid showed tubular epithelial structures, as confirmed using bright-field periodic acid-Schiff (PAS) staining, and confocal microscopic analysis confirmed the presence of six to ten nephrons (Fig. 1B; Fig. S1A-D). These nephrons showed evidence of early patterning and segmentation. The formation of glomeruli was evident from positive staining for NPHS1 and MAFB (Fig. 1B,C; Fig. S1B-D). Proximal nephron segments were EpCAM^+^ and stained positive for *Lotus tetragonolobus* lectin (LTL), CUBN, LRP2 and HNF4A (Fig. 1B,C; Fig. S1B-D). LTL^+^ segments were able to endocytose fluorescein isothiocyanate (FITC)-albumin within 24 h of addition to the culture medium, which indicated a functional albumin uptake pathway (Fig. S1E). Distal nephron segments were stained with ECAD (CDH1) and EpCAM, whereas a presumptive collecting duct/connecting segment was ECAD^+^/GATA3^+^ (Fig. 1B,C; Fig. S1B,C). The presence of endothelial cells (PECAM1^+^/SOX17^+^) (Fig. 1C) was also noted when kidney micro-organoids were generated using a *SOX17mCherry* reporter cell line (Ng et al., 2016) (Fig. S1C,D). As an indication of the transferability of the protocol between hPSC lines, we provide data on the successful generation of kidney micro-organoids from four different cell lines, including hESC reporter lines (H9 GAPTrap*Luc2*, hES3-*SOX17mCherry*) (Ng et al., 2016; van den Berg et al., 2018; Kao et al., 2016) and human iPSCs (CRL1502.C32, CRL1502.3) (Briggs et al., 2013; Takasato et al., 2015). All hPSC lines uniformly responded to the protocol and patterned similarly to kidney micro-organoids (Fig. S1F).

### Effect of duration, concentration and timing of canonical Wnt signalling on kidney micro-organoid patterning

We have previously demonstrated that the initial duration of CHIR99021 induction can result in a shift in primitive streak patterning such that the resulting IM is specified to be more or less anterior (Takasato et al., 2015). To optimise differentiation within the kidney micro-organoid protocol, monolayers of hPSCs were stimulated using a fixed concentration of CHIR99021 (7 µM) for varying durations (3, 4, 5 and 6 days) before continued culture to day 7 in the presence of low CHIR/FGF9/heparin (Fig. S1G-I). After 18 days, the resulting micro-organoids were evaluated for kidney structure using confocal microscopy (Fig. S1G-I). Canonical Wnt activation for only 3 days failed to generate a kidney morphology (Fig. S1G-I, left panel). Instead, the epithelial structures that were present exhibited an undefined epithelium with a large cystic lumen and no evidence of nephron formation. Initial induction with 4 or 5 days of CHIR99021 generated micro-organoids that contained patterning nephrons, including the presence of surrounding SOX17^+^ and MEIS1/2/3^+^ populations, which suggested the presence of endothelium and interstitial stromal cells, respectively (Fig. S1G-I, middle panels). However, Wnt activation for 6 days, although generating larger kidney micro-organoids with greater NPHS1 staining, contained an expanded MEIS1^+^ stromal population of apparent epithelial structure (Fig. S1G-I, right panel). Subsequent cultures were performed using 4 days of initial CHIR99021 induction, as per Takasato et al. (2016).

The inclusion of low levels of CHIR99021 together with FGF9 from day 4 of monolayer culture was a deviation from our previous protocol that was prompted by published evidence that low levels of canonical Wnt signalling support self-renewal of nephron progenitors (NP) (Karner et al., 2011). Immunofluorescence for the IM/nephron progenitor/nephron marker PAX2 was clearly higher in day 7+11 kidney micro-organoids exposed to 1µM CHIR99021 (Fig. 1D), with qPCR performed on the day 7 monolayer cultures showing increased expression of key IM/nephron progenitor markers (*PAX2*, *WT1*, *LHX1*, *OSR1*, *SIX1*, *EYA1*) as well as *WNT11* and *GATA3*, which are expressed in the ureteric tip and collecting duct but are not unique to these sites (Fig. 1E) (Majumdar et al., 2003; Grote et al., 2006; Takasato et al., 2015).

### Transcriptional validation of kidney differentiation within micro-organoids

**Fig. 2.**
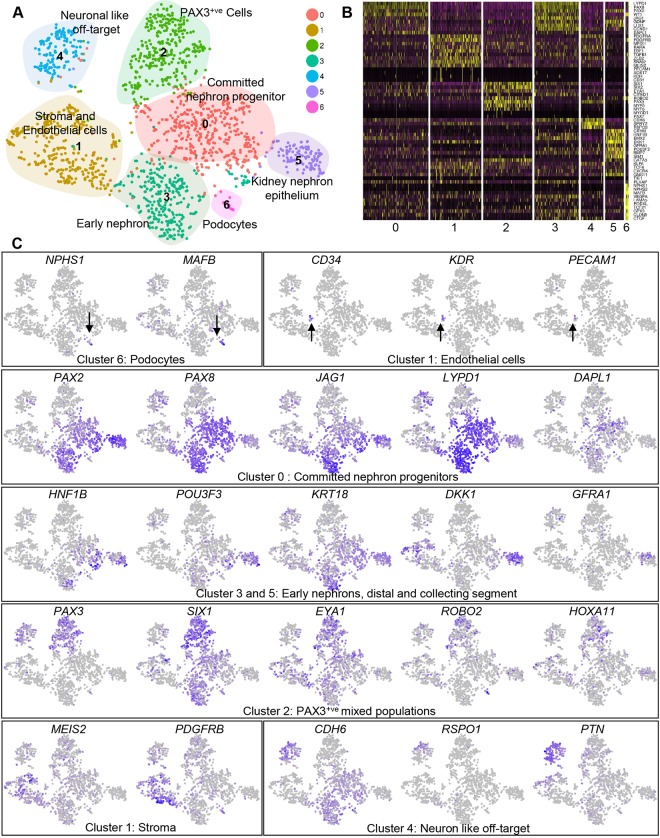
**Transcriptional validation of kidney differentiation within micro-organoids.** (A) *t*-SNE plot after Seurat clustering of single cell RNA-seq of day 7+18 CRL1502-C32 micro-organoids showing 7 different clusters. (B) Heat-map showing scaled gene expression of key marker genes within clusters. (C) *t*-SNE plots indicating the expression of key marker genes for selected nephron cell types. Colour intensity is scaled per gene, darker blue indicates higher expression. Arrows indicate podocytes and endothelial cells.

Characterisation of the cell types that are present within kidney micro-organoids was performed using single cell RNA-sequencing (scRNA-seq). A pool of 20-30 micro-organoids was dissociated into viable single cells using cold active protease Liberase. This resulted in the generation of 89.4% single cells, out of which 88.5% cells were live (data not shown). Cell Ranger (10x Genomics) was used to generate a matrix of unique molecular identifier (UMI) counts per cell which was imported into for further analysis using the Seurat R package (version 2.3.1) (Satija et al., 2015). Data on the number of UMIs per cell, proportion of mitochondrial gene expression and predicted cell cycle state (Scran 1.6.7) (Lun et al., 2016) by cluster is represented in Fig. S2A,B. Filtered data represented 1673 cells with a median of 3759 expressed genes per cell. Clustering using the Seurat R package produced seven distinct cell clusters (Fig. 2A; Fig. S2A-D) at 0.6 resolution. Differential expression testing was performed to identify markers of each cluster (Table S1), and Gene Ontology and functional enrichment analysis for the top significantly upregulated genes in each cluster was performed using the PANTHER Gene Ontology suite (Mi et al., 2013) (Fig. S2D). Although clearly evident using immunofluorescence of whole-mount organoids, the endothelium (a subset of Cluster 1) and podocytes (Cluster 6, 18 cells) were represented by only small numbers of individual cells in the scRNA-seq data. Cluster 3 (293 cells) and Cluster 5 (122 cells) showed expression of genes that was consistent with kidney nephron epithelium, with Cluster 0 showing expression of renal vesicle/S-shaped body genes (early nephron), whereas epithelial cell Cluster 5 also showed expression of distal tubule/collecting duct markers such as *GFRA1* (Fig. 2B,C; Fig. S2E). Cluster 2 showed expression of the nephron progenitor markers *SIX1*, *SIX2* and *CITED1*, as well as the stromal marker *PAX3* that has previously been associated with myogenic Wilms' tumours (Hueber et al., 2009). Cells in Cluster 2 also expressed markers of myogenic fate such as *MYF5* and *MYF6*, but not *PAX7*, *MYOD1* and *TBX6*. Cluster 0 (430 cells), the largest cluster, showed a more committed nephron progenitor signature, with expression of early renal vesicle markers *PAX2*, *PAX8*, *LHX1* and *JAG1* as well as the human NP markers *LYPD1* and *DAPL1* (Lindstrom et al., 2018). Cluster 1 (337 cells) showed a stromal signature, with the expression of *PDGFRB* and *MEIS2*. Cluster 4, although expressing the early nephron marker *CDH6*, showed a neural transcriptional signature, which suggested the presence of a neural off-target population, as has been previously reported in kidney organoids (Wu et al., 2017 preprint). This analysis strongly supported the identity of the cell types observed within kidney micro-organoids at the level of immunofluorescence.

### Comparative single cell transcriptional profiling of standard and micro-organoids demonstrates equivalence of nephrogenic differentiation

**Fig. 3.**
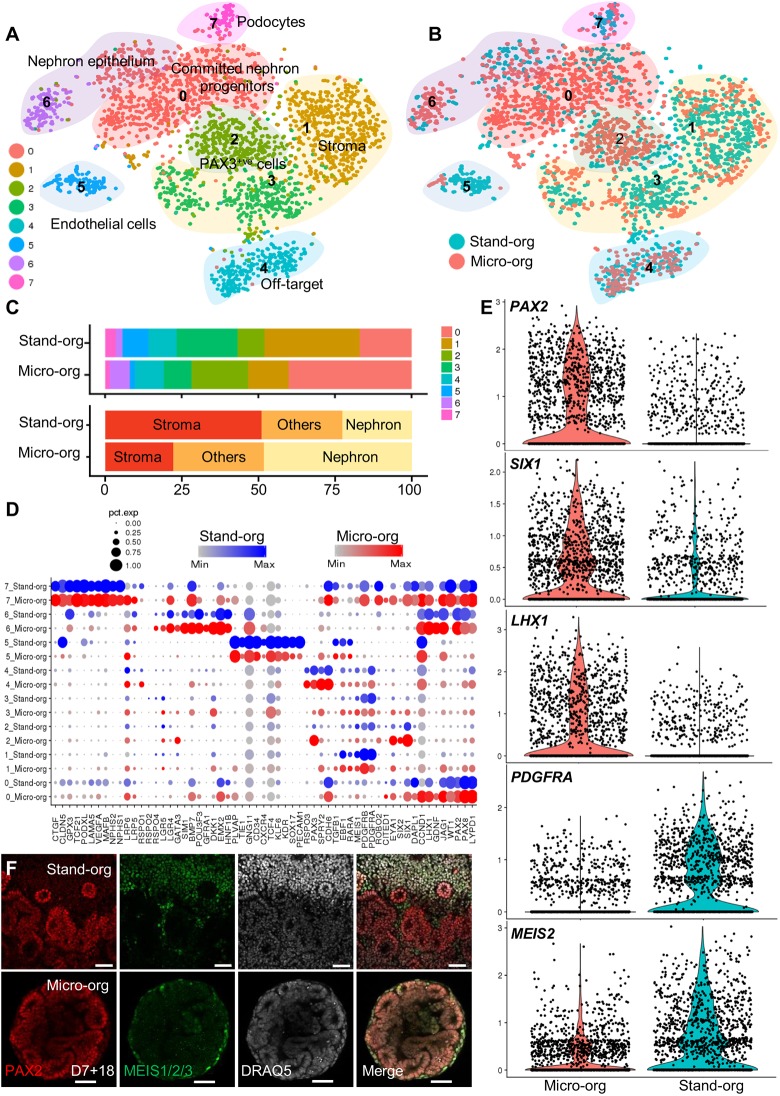
**Comparative single cell transcriptional profiling of standard kidney organoids and micro-organoids demonstrates an equivalent nephrogenic patterning.** (A) *t*-SNE plots after integrated Seurat analysis of kidney micro-organoid and standard organoid 10x scRNA-seq data from day 7+18 (CRL1502.32 cells) (Combes et al., 2019). (B) *t*-SNE plot representing micro-organoid (Micro-org; pink) and standard organoid (Stand-org; blue) contributions to cell types in each cluster. (C) Bar graph representing the proportion of each of the Micro-org or Stand-org datasets assigned to each transcriptional cluster and differentiation lineage type. (D) Split dot plots showing the gene expression of kidney markers in each cluster between kidney micro-organoids and standard organoid. (E) Violin and scatter plots showing the log-normalised counts per cell for nephron-related (PAX2, SIX1, LHX1) and stromal-related (PDGFRA, MEIS2) genes within Micro-org and Stand-org. (F) Immunofluorescence showing the expression of PAX2 and MEIS1/2/3 between kidney Micro-org and Stand-org. Scale bars: 50 µm.

In order to compare directly the cellular components within kidney micro-organoids to another kidney organoid method, we overlaid the micro-organoid scRNA-seq data with standard kidney organoid scRNA-seq data (1421 kidney organoid cells) that were generated simultaneously using the same iPSC cell line (CRL1502.C32). The two datasets were combined using the alignment algorithm implemented in Seurat (Butler et al., 2018), which uses correlated component analysis followed by dynamic time warping. Data on the number of UMIs per cell, the proportion of mitochondrial gene expression and the predicted cell cycle state (Scran 1.6.7) by cluster is represented in Fig. S3A-C. Clustering identified eight transcriptional clusters within the combined dataset, which represents committed nephron progenitors (Cluster 0), nephron epithelium (Cluster 6), podocytes (Cluster 7), stroma (Clusters 1 and 3), endothelial cells (Cluster 5), PAX3^+^ cells (Cluster 2) and a neural off-target population (Cluster 4) (Fig. 3A; Figs S3D,E and S4A). All clusters were represented in both datasets, though the proportion of cells that was attributed to each cluster varied (Fig. 3B,C). A direct comparison of key markers of each cluster shows that, although there were apparent differences in the proportion that contributed to each cluster between protocols (Fig. 3B,C), there was strong transcriptional congruence between the cells that were identified in any given cluster between both protocols (Fig. 3D). The neural off-target population that was identified in kidney micro-organoids was also evident in standard organoids. Overall, the micro-organoid dataset contained a higher proportion of nephron cells and a lower proportion of stromal cells than the standard organoid dataset (Fig. 3E; Figs S3D and S4B). This increase in PAX2-expressing nephron cells and the reduction in MEIS1/2/3-expressing stromal cells in micro-organoids compared with standard organoids was confirmed using immunofluorescence analysis of whole-mount organoids (Fig. 3F).

### Kidney micro-organoids provide a platform for efficient hPSC-derived kidney cell scale-up

**Fig. 4.**
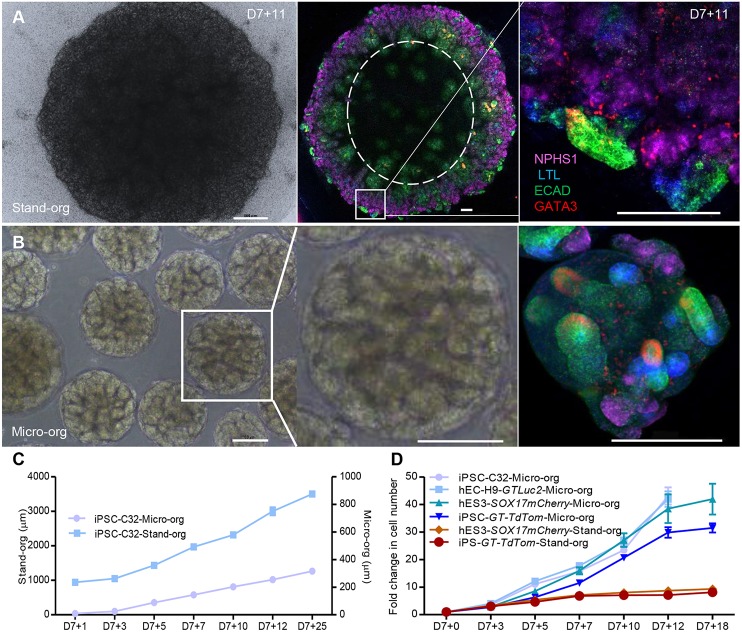
**Kidney micro-organoids provide a better platform for efficient hPSC-derived kidney cell scale-up.** (A) Bright-field image of standard kidney organoid at day 7+11 (left), confocal immunofluorescence image (tile scan) of entire standard organoid showing the spatial restriction of nephron structures to the edge of the organoid (middle), and magnified image of the boxed area showing a nephron within that organoid (right). (B) Bright-field image of kidney micro-organoids (left) and magnified bright-field image of the boxed area, showing a single kidney micro-organoid (middle), and confocal immunofluorescence image of kidney micro-organoids at day 7+11 (right). (C) Change in size of the organoids at different stages of development. (D) Fold change in cell number (and scalable capacity) of micro-organoids compared with standard organoid over time. Scale bars: 500 µm (A, left); 200 µm (A, middle and right, B).

Standard kidney organoids that are cultured on Transwell filters may face diffusion limitations after 3 weeks in culture due to the size of the organoid tissue generated (Fig. 4A; Fig. S4C). Immunofluorescence staining for the nephron segments after this time suggested a spatial restriction of nephron structures to the edge of the organoids. By way of contrast, kidney micro-organoids contain kidney tubules throughout the structure (Fig. 4B). Kidney micro-organoids can also be formed simultaneously in large numbers using an orbital shaker, which avoids the tedious process of manual handling that is involved in the standard organoids protocol. As a result, it is possible to generate approximately 8000 to 10,000 kidney micro-organoids of uniform size in 5-10 min compared with approximately 30 organoids in 60 min for standard organoid protocol. Kidney micro-organoids exhibit much smaller final size (250-300 μm) compared with standard organoids (3000-5000 µm) (Fig. S4C,D). As shown by immunofluorescence, the nephrons that form within a standard organoid are present in a rim around the periphery of the tissue in comparison with the kidney micro-organoids (Fig. 4A). However, these structures are much larger than the micro-organoids. In order to directly compare the efficiency and cost of each approach, standard organoids and kidney micro-organoids, each generated using iPSC and hESC reporter lines, were dissociated to a single cell suspension at multiple time points across the differentiation protocol from day 7 for quantification of total cell number (Fig. 4C,D). Standard organoids did not show a substantive change in total cell counts per organoid after day 7+7, whereas micro-organoids continued to increase in total cell number until day 7+12. Overall, cell count increased eight- to tenfold under standard organoid conditions but up to 30- to 40-fold in the case of kidney micro-organoids. This represents a three- to fourfold improvement in cell yield using this modified protocol. When considering a formal comparison of cell yield and cost per million cells (Table 1), the use of the kidney micro-organoid culture format reduced cost per million cells by 75%.

**Table 1.**
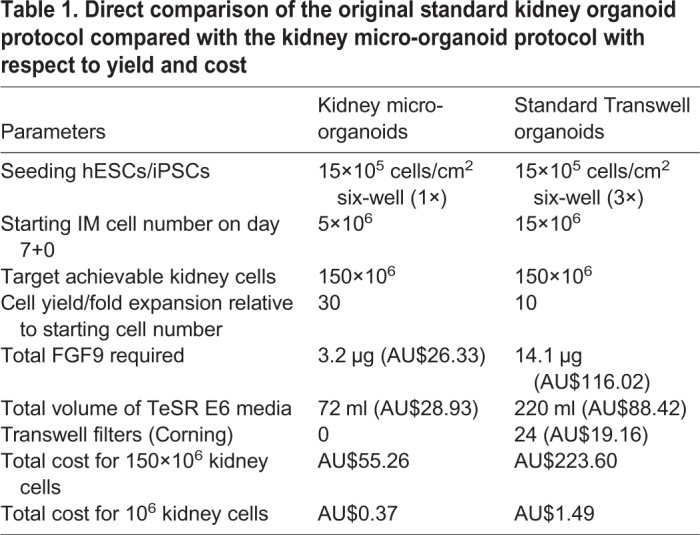
**Direct comparison of the original standard kidney organoid protocol compared with the kidney micro-organoid protocol with respect to yield and cost**

### Extended micro-organoid culture

**Fig. 5.**
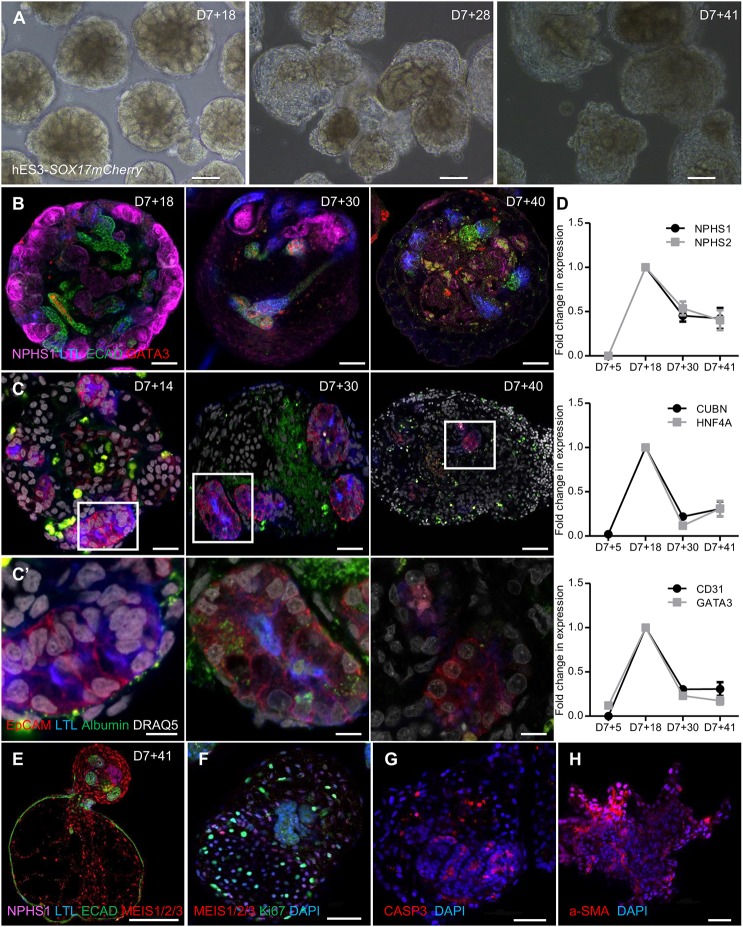
**Extended micro-organoid culture.** (A) Bright-field images of extended culture of kidney micro-organoids in suspension using hES3-*SOX17 mCherry* cells on day 7+18, day 7+28 and day 7+41. (B) Confocal immunofluorescence images of different nephron segments on day 7+18, day 7+30 and day 7+40. (C,C′) Confocal immunofluorescence images showing albumin (FITC) uptake at different stages of micro-organoid culture. C′ shows magnified images of the boxed areas in C above. (D) qPCR showing the fold change in gene expression for different nephron segments on day 7+5, day 7+18, day 7+30 and day 7+41 of kidney micro-organoid culture (*n*=3). Top, podocytes; middle, proximal tubules; bottom, distal tubules and endothelial cells. (E-H) Confocal immunofluorescence images of hES3-*SOX17 mCherry* micro-organoids after extended culture to day 7+41, illustrating morphological changes. Dysplastic organoids are stained for different segments of nephron and stoma (E), show proliferation within an expanding stromal population (F), and evidence of apoptotic cells (CASP3^+^) (G) and fibrotic (α-SMA) lesions (H). Scale bars: 100 µm (A); 50 µm (B,E-H); 20 µm (C); 5 µm (C′).

Whereas micro-organoids showed a superior cell yield until day 7+25 of culture, the formation of variable patterns of dysplasia, including cyst formation and/or mesenchymal expansion, was present in suspension culture from day 7+28 onwards (Fig. 5A,B; Fig. S5A,B). The resulting histology clearly varied with cell line, which suggests that extended culture of micro-organoids in suspension is suboptimal. Other protocols that report the development of kidney organoids in suspension culture have described similar pathology (Cruz et al., 2017; Czerniecki et al., 2018; Przepiorski et al., 2018), and attribute this variably to mutational status, fibrotic expansion or a consequence of detachment and suspension culture itself. This phenomenon of cyst formation was previously described as a feature of iPSC lines that had been modified using CRISPR/Cas9 gene editing to carry homozygous mutations of the PKD1 genes (Cruz et al., 2017). In contrast, we show that this is a feature of prolonged suspension culture even using a number of distinct wild-type human iPSC (CLR1502.3) and hESC (hES3-*SOX17mCherry* and H9 *GAPTrap-Luc2*) lines. Extended culture of micro-organoids also resulted in reduced albumin uptake, which indicates the loss of functional proximal tubule by day 7+41 (Fig. 5C,C′). Conversely, albumin uptake is suboptimal at earlier stages, such as day 7+14 (Fig. 5C,C′), which suggests that proximal tubules are not sufficiently mature at that time point. qPCR analysis across time confirmed these conclusions, showing reduced expression of many kidney marker genes, including the proximal tubule markers *CUBN* and *HNF4A* (Fig. 5D). Immunofluorescence analysis of day 7+41 hES3-*SOX17mCherry* micro-organoids suggested the expansion of MEIS1^+^ stromal cells and a loss of tubular epithelium, with evidence for Ki67 staining in the stromal compartment and evidence of apoptosis of the epithelium (CASP3^+^), followed by extracellular matrix (α-SMA) deposition that resulted in fibrotic lesions (Fig. 5E-H). All of the above changes contribute to a loss of epithelial tubular structures within the micro-organoids, which further limits the utility of extended micro-organoid culture in suspension. This would suggest that although accurate patterning of nephrons can be initiated using this format of culture, prolonged culture is not an effective means to mature such structures and application needs to be timed with optimal nephron identity. However, the enhanced expansion of cell number using this approach provides an initial advantage with respect to yield.

### Adriamycin-induced toxicity in micro-organoids

**Fig. 6.**
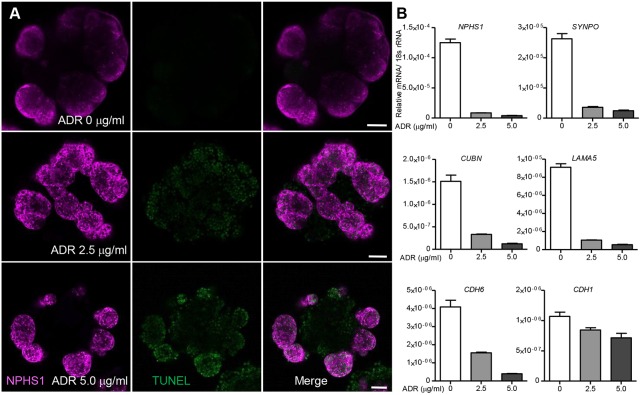
**Adriamycin-induced toxicity in micro-organoids.** (A) Confocal immunofluorescence image analysis of micro-organoids after treatment with Adriamycin (0, 2.5 and 5 µg/ml) for 24 h. Apoptotic cells were identified by TUNEL staining. (B) qPCR analysis showing dose-dependent toxicity induced by Adriamycin on kidney organoids by reduced expression for kidney marker genes (*n*=2). Scale bars: 50 µm.

Although this approach can be used as a means to generate large numbers of early nephron cell types, it may also prove an effective approach for low-cost high-content evaluation of toxicity or drug efficacy. In order to evaluate the utility of micro-organoids for assessing drug toxicity, kidney micro-organoids were treated with different doses of Adriamycin, a known nephrotoxic antibiotic, for 24 h in a 24-well plate format. Podocytes within the kidney micro-organoids exhibited TUNEL positivity within the podocyte compartment after the Adriamycin treatment (Fig. 6A). These results were confirmed using qPCR analysis (Fig. 6B), which showed a dose-dependent reduction in the expression of nephron segment markers, particularly those marking podocytes. This pilot evaluation confirms the feasibility of using kidney micro-organoids for drug toxicity screening.

## DISCUSSION

In this study, we describe a modified protocol for the generation of human kidney organoids from pluripotent stem cells that involves minor modifications during initial IM patterning, aggregate formation and subsequent culture format. The resulting kidney micro-organoids show reliable formation of kidney nephron epithelial, stromal and endothelial cellular components that are equivalent at a single cell transcriptional level to those present within our previously described kidney organoid protocol (Takasato et al., 2015, 2016). However, the alterations in culture conditions resulted in a three- to fourfold improvement in relative cell yield at a 75% lower cost per million kidney cells generated. The robustness of this protocol is evidenced by our capacity to recapitulate successful micro-organoid generation using two different hESC (H9 and hES3) and three different iPSC lines (iPSC GAPTrap td-Tomato, CRL1502.C32 and CLR1502.3), including hES3 *SOX17mCherry*, H9 GAPTrap *Luc2* and iPSC GAPTrap *td-Tomato* fluorescent reporter lines.

In the kidney organoid field, as in the pancreas and brain organoid fields, there is a growing number of protocols which report the formation of kidney structures *in vitro*. In this report, the micro-organoid protocol was only directly compared here with the differentiation method of Takasato et al. (2015). A prior protocol described by this group (Takasato et al., 2014) did not require any aggregation. Instead, cultures were left as monolayers with the spontaneous formation of small clusters of kidney cells across the field of culture. This is more akin to the protocol of Freedman et al. (2015), which results in substantial areas of non-renal cells within each well. Such approaches appear to be less targeted in their specification. Another recent report has identified a similar bioreactor-based low-cost approach for the generation of kidney organoids in suspension culture (Przepiorski et al., 2018). In that method, the only specifying factor was the addition of CHIR99021 to embryoid bodies from the commencement of culture, which was followed by the subsequent selective enrichment of kidney-containing organoids based on sphere size. This approach may generate less reliable patterning given the lack of evidence of initial IM patterning in all structures. The micro-organoid protocol described here has the advantage of specifying to IM before aggregate formation. Based on our single cell transcriptional profiling, the component cell types in micro-organoids align very closely to what is occurring in the Takasato et al. protocol (Takasato et al., 2015), although there is some evidence of enrichment for a previously identified neural off-target subpopulation (Combes et al., 2017 preprint; Wu et al., 2017 preprint). The identity of cellular diversity that is present within the organoids described by Przepiorski et al. (2018) was not fully defined.

The presence of a pathological fibrotic response within organoids in suspension culture was also observed in the study of Przepiorski et al. (2018). It was proposed that this may represent a model of interstitial fibrosis as occurs in certain postnatal disease settings. Given the immaturity of the cell types within hPSC-derived kidney structures, we would suggest that it may be too optimistic for this to represent an accurate model of a postnatal event. Indeed, we show a variable pathology that depends upon the line in use (Fig. S5). Overall, this suggests limited utility of suspension culture beyond approximately 30 days of expansion, which coincides with a cessation of cell expansion (Fig. 4D). The variability in eventual morphology between lines may reflect differences in relative levels of off-target population, which is in turn influenced by the initial patterning to intermediate mesoderm, the size of the organoid and the number of organoids in the culture well. In other protocols for the generation of iPSC-derived organoids, such as cerebral organoids (Lancaster et al., 2013), the initial patterning phase is supported by a media that is distinct from that used for later maturation conditions. It is likely that it will be necessary to define such conditions to allow prolonged tubular survival and further differentiation. As noted, even long-term culture of standard kidney organoids on Transwell plates does not result in continued nephron formation or maturation (van den Berg et al., 2018). This does not reduce the value of the improved cell expansion of this suspension culture approach, which would be amendable for the production of large cell numbers for use in cellular therapy or tissue engineering.

In conclusion, this modified culture format for the accurate and reliable differentiation of human iPSCs to kidney micro-organoids will facilitate substantial expansion of renal cell types, which will improve utility for cell therapy and tissue engineering.

## MATERIALS AND METHODS

### Culture and maintenance of hPSC

hESCs (H9 and hES3 cells) were grown on mouse embryonic fibroblast (MEF) feeders in Dulbecco's Modified Eagle's medium (DMEM) supplemented with 10% KnockOut Serum Replacement (KOSR; Life Technologies) and 20 ng/ml bFGF (R&D Systems). Before the initiation of differentiation, ESCs were adapted to a Matrigel (Corning) surface without feeders in an MEF conditional media and 20 ng/ml bFGF. Human iPSCs were grown on Matrigel-coated plates using E8 media (Life Technologies) once the cells reached 60-70% confluence, or every 3 days once cells were detached using EDTA and cell clumps reseeded onto fresh Geltrex-coated plates (Life Technologies).

### hPSC differentiation and generation of kidney micro-organoids

Initially, hPSC were differentiated into IM using a modified Takasato's protocol (Takasato et al., 2016, 2015). Briefly, hPSC were dissociated into single cells using TrypLE Select and seeded on to Matrigel-coated plates at a density of 15,000 cells/cm^2^ using MEF conditional media or E8 media with Revitacell (Thermo Fisher Scientific), and left to adhere overnight at 37°C in a standard cell culture incubator. Differentiation into kidney organoids involves two steps. Step 1: Primitive streak was induced by treating 2D monolayer cultures of hPSCs with 7 µM CHIR99021 (Tocris Bioscience) for 4 days in TeSR-E6 media (Stemcell Technologies). PS cells were driven into IM from day 5 to day 7 by treating with 200 ng/ml FGF9, 1 µg/ml heparin and 1 µM CHIR99021. This resulted in the induction of a mixture of IM cells. Step 2: To form micro-organoids, IM cells were washed with 1 ml of 0.1 M PBS on day 7 and then cells were dissociated using either 1.5 ml of EDTA or TrypLE Select for 3 min at 37°C. Dissociated cells were washed with plain DMEM and centrifuged at 0.4 ***g***. The cell pellet was resuspended in 2 ml of Stage 1 media [base media containing 200 ng/ml FGF9, 1 µg/ml heparin, 1 µM CHIR99021, 0.1% PVA, 0.1% MC, 10 µM Rho kinase inhibitor (Tocris Bioscience)]. The cell suspension was transferred to 6 cm^2^ low adhesion plates (Greiner Bio-One). Organoids spontaneously formed after placing the culture dishes on an orbital shaker (Ratek) rotating at 60 rpm in a standard cell culture incubator at 37°C and 5% CO_2_. After 24 h. Stage 1 media was replaced with Stage 2 media (base media containing 200 ng/ml FGF9, 1 µg/ml heparin, 1 µM CHIR99021, 0.1% PVA, 0.1% MC) for another 4 days. From day 7+5 onwards all organoids were refreshed with Stage 3 media (base media containing 0.1% PVA, 0.1% MC) and continued until day 7+18 or the desired end point by refreshing on alternative days.

### Dissociation of kidney micro-organoids

Micro-organoids represent a heterogeneous epithelial structure, approximately 250-300 µm in diameter. Use of harsh enzymes may destroy cell-surface markers, which would lead to the loss of cell identity for later use. Mild dissociation with a cold active protease (Liberase, Roche) was performed to yield maximum viable single cells. Micro-organoids were transferred to a 15 ml falcon tube using 5 ml serological pipette and allowed to settle. The supernatant was removed using vacuum, and the organoid pellet was washed 3× using 0.1 M PBS. The organoids were then treated with 500 µl of a 1 µg/ml solution of Liberase and incubated at 4°C for 20 min with continued trituration using a p1000 Gilson pipette every 5 min. After 20 min micro-organoids had dissociated into single cells, and were washed twice using DMEM with 10% foetal calf serum (FCS) to inactivate the Liberase. The final cell pellet was suspended in DMEM with 10% FCS.

### Single cell RNA analysis of standard and micro-organoids

Approximately 40-50 kidney micro-organoids and one entire standard organoid were cultured to day 7+18 using the same hPSC line (CRL1502.C32 in APEL media). Organoids were dissociated using Liberase for 20 min at 4°C. Cells were centrifuged at 0.4 ***g*** for 3 min to form a pellet. Single cells were resuspended in fresh DMEM F12 media filtered through 20 µm cell strainers to remove clumps, and stored on ice until analysis. Viability and cell number were analysed using a Trypan Blue dye exclusion test in an automated cell counter (Life Technologies). Cells were thoroughly mixed using a wide bore 1 ml pipette tip and approximately 4000 live cells were taken for the analysis. Sample preparation was carried out according to the 10x Genomics single cell protocol. Briefly, cells were barcoded to index separately the transcriptome of each cell using nanolitre-scale Gel Bead in Emulsions (GEMs) and UMIs. Magnetic beads were used to remove leftover reagents and primers after barcoding. Full-length barcoded cDNA was then amplified using PCR to generate sufficient mass for library construction. These libraries were sequenced simultaneously for UMIs and cDNA fragments using paired-end reads. Library analysis was performed using Cell Ranger. The Cell Ranger pipeline (v1.3.1) was used to perform sample demultiplexing, barcode processing and single cell gene counting (Zheng et al., 2017). Samples were demultiplexed to produce a pair of FASTQ files for each sample. Reads containing sequence information were aligned to the GRCh38 reference genome. Cell barcodes were filtered to remove empty droplets and PCR duplicates were removed by selecting unique combinations of cell barcodes, UMIs and gene IDs, with the results being a gene expression matrix that was used for further analysis. Further analysis was performed to represent cell clustering, cell type classification and differential gene expression using the Seurat R package (version 2.3.1) (Satija et al., 2015).

Gene expression matrices generated in Cell Ranger were imported into Seurat (Satija et al., 2015) for quality control and further analysis. The cyclone function in Scran (Lun et al., 2016; Scialdone et al., 2015) was used to assign a score that was related to the likelihood that each cell is in either G1, S or G2M phase, and a cell cycle phase was assigned based on this scoring. Initial filtering removed genes that were expressed in fewer than three cells, and cells with fewer than 200 genes expressed. Expression data was normalised and scaled, with variability related to the number of UMIs, percentage mitochondrial expression and G2M, S and G1 score regressed out using the Seurat ScaleData function. Cells were clustered using the shared nearest neighbour modularity optimisation-based clustering algorithm implemented in Seurat using the first 20 principal components and a resolution of 0.6. Marker gene lists were generated using the Seurat FindAllMarkers function to find differentially expressed genes between clusters, with a log fold change above 0.25. Differential expression testing was performed and Gene Ontology and functional enrichment analysis for the top significantly upregulated genes in each cluster was performed using the PANTHER Gene Ontology suite (Mi et al., 2013).

For combined analysis of standard and micro-organoid datasets, gene-cell matrices were generated in Cell Ranger as above. Each dataset was normalised and scaled with regression against the number of UMIs, percentage mitochondrial expression and an S, G1 and G2M score was generated in Scran. Clustering was based on the first 20 aligned combined components calculated in Seurat using the RunCCA and AlignSubspace functions (Butler et al., 2018). For the combined dataset, clustering was performed at a resolution of 0.6.

### Kidney micro-organoids size and total cell number measurement

Organoid size (diameter) was measured using bright-field images of up to ten randomly sampled organoids on NIS-Elements microscopy software (Nikon). Organoid size was reported as a low to high range. To quantify the total number of cells, organoids were suspended in 5 ml of media and three random 50 µl samples were collected. The organoids were dissociated into single cells using TrypLE Select and the total number of cells was counted manually using a haemocytometer, with the results extrapolated to 5 ml.

### Albumin uptake assay

Kidney micro-organoids were generated as previously described. Albumin uptake was determined at different time intervals (day 7+14, day 7+18, day 7+30 and day 7+40) by treating organoids with FITC-labelled albumin for 24 h in a 24-well low adhesion plate on an orbital shaker. After 24 h organoids were washed 10× with 1× PBS before fixing with 2% paraformaldehyde (PFA). Then the albumin uptake was analysed by immunostaining with LTL, EpCAM and nuclear staining (DRAQ5) followed by confocal microscopic analysis.

### Real time-qPCR analysis

Organoids were lysed in lysis buffer and mRNA was isolated from organoids using the Ambion mRNA Isolation Kit (Life Technologies) according to the manufacturer's instructions. We converted 0.5-1 µg mRNA to cDNA in the presence of thermostable RNAse inhibitor and a reaction mix containing GoScript reverse transcriptase (Promega), MgCl_2_, nucleotide mix and 5× reaction buffers, using standard GoScript reverse transcription protocol. RT-qPCR analysis was performed using GoTaq polymerase and the SYBR Green detection system (Promega) using the Applied Biosystems 7500 Real-Time PCR System with the help of the human gene-specific primers listed in Table S2.

### Immunofluorescence staining of kidney micro-organoids

Kidney micro-organoids were collected in a 15 ml flacon tube and washed 2× with PBS to remove excess media and fixed in freshly prepared 2% PFA for 20 min at 4°C. Excess PFA was then removed by washing the organoids 3× with PBS with 0.3% Triton X-100 (PBST) and stored in PBST at 4°C until staining. Fixed organoids were blocked in 10% donkey serum in PBST (blocking buffer) for at least 1 h before incubation with primary antibodies that were diluted in blocking buffer. Evaluation of the differentiation capacity of micro-organoids was confirmed by staining for major nephron segments. The primary antibodies used were: sheep anti-human NPHS1 (1:300, AF4269, R&D Systems), biotin anti-human LTL (1:300, B-1325, Vector Laboratories), mouse anti-human ECAD (1:300, 610182, BD Biosciences), rabbit anti-human GATA3 (1:300, 5852S, Cell Signaling Technology), mouse anti-human CD31 (1:300, 555444, BD Biosciences), goat anti-human CUBN (1:300, sc-20607, Santa Cruz Biotechnology) and rabbit anti-human LRP2 (1:300, NBP2-39033, Sapphire Bioscience). Organoids were incubated in primary antibodies overnight at 4°C, washed 5× in PBST, and then incubated with species-matched secondary antibodies with fluorescent labels. After the staining, organoids were dehydrated using a methanol series (25%, 50%, 75% and 100% for 5 min each) followed by clearing using benzyl alcohol and benzyl benzoate (BABB, 1:2 ratio) as previously described by Dodt et al. (2007). Cleared organoids were mounted on a glass-bottom dish (MatTek Corporation) and confocal microscopy was performed using an inverted LSM 780 microscope (Zeiss) with a 25× multi-immersion objective. The images were analysed using ZEN software (Zeiss).

### Statistical analysis

Data are represented as mean±s.e.m. The comparison between the groups was calculated using an unpaired *t-*test (two-tailed) and a value of *P*<0.05 was considered to be statistically significant. All statistical analyses were carried out using GraphPad Prism software (version 5).

## Supplementary Material

Supplementary information
