# Supplementary material for: Kidney micro-organoids in suspension culture as a scalable source of human pluripotent stem cell-derived kidney cells
Source: Development. 2019 Mar 7;146(5):dev172361. doi: 10.1242/dev.172361 (PMC6432662; doi:10.1242/dev.172361)
Supplement: Supplementary information [file develop-146-172361-s1.pdf]

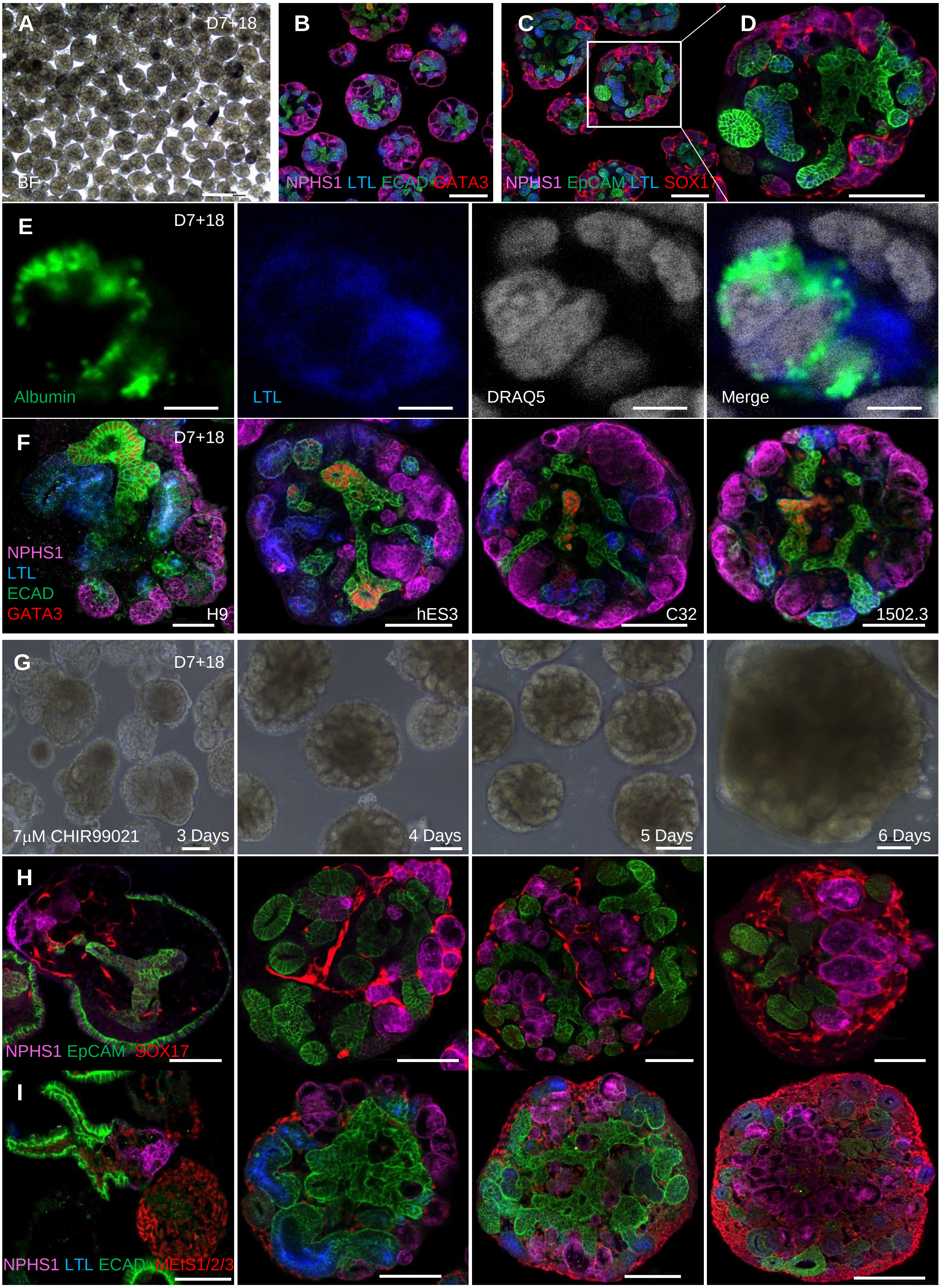
A
D7+18
BF
B
NPHS1 LTL ECAD GATA3
C
NPHS1 EpCAM LTL SOX17
D
E
D7+18
Albumin
LTL
DRAQ5
Merge
F
D7+18
NPHS1
LTL
ECAD
GATA3
H9
hES3
C32
1502.3
G
D7+18
7µM CHIR99021
3 Days
4 Days
5 Days
6 Days
H
NPHS1 EpCAM SOX17
I
NPHS1 LTL ECAD MEIS1/2/3

Fig. S1: Generation of micro-kidney organoids in suspension culture and initial Wnt signalling is important for kidney organoid development and maturation. **(A)** Bright field images showing uniform kidney micro-organoids on Day 7+18 (scale 500 μm). **(B, C and D)** Immunofluorescence and confocal images of micro-organoids showing formation of nephron segments independent of micro-organoid size and shape, including development of vascular structures (scale 100 μm). **(E)** Confocal image of micro-kidney organoid tubules on Day 7+18 showing FITC albumin uptake (scale 5μm). **(F)** Confocal images of micro-kidney organoids generated using 4 different cell lines, including hES (H9 GAPTrap Luc2, hES3 SOX17mCherry) and iPS (CRL1502.C32 and CRL1502.3) on Day 7+18 with antibodies labelling different nephron segments (scale 50 μm). (**G, H and I)** hES3 SOX17mCherry derived micro-organoids generated after exposure to different days of initial 7 μM CHIR99021 for 3, 4, 5 and 6 days treatment showing bright field (G, scale 100 μm) and immunofluorescence confocal images showing SOX17+ vasculature **(H)** and MEIS1/2/3+ stroma **(I)** (scale 100 μm)

Development • Supplementary information

Development: doi:10.1242/dev.172361: Supplementary information

A

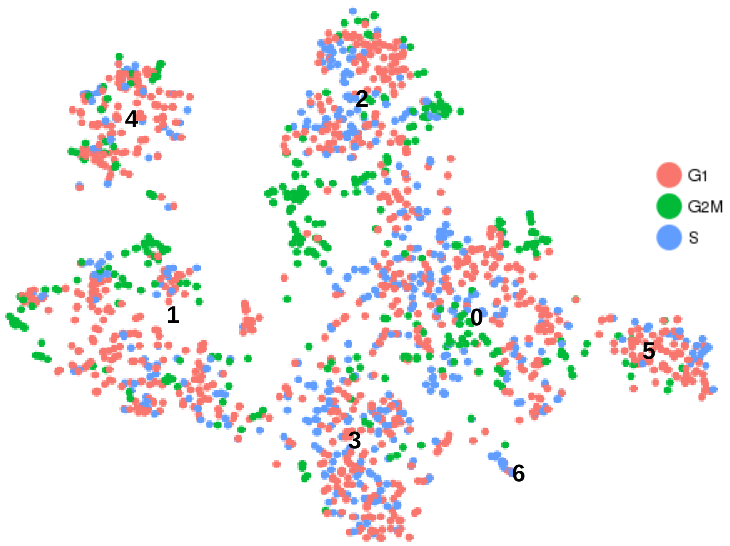

B

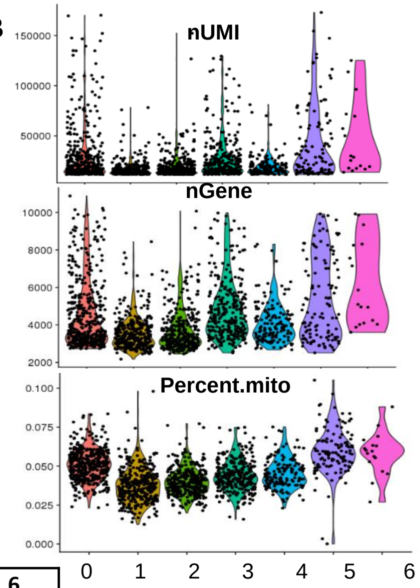

C

Cluster	0	1	2	3	4	5	6
No. of Cells	430	337	324	293	149	122	18

D

Cluster	GO biological process complete	Go Term	Fold Enrichment	FDR
0	pattern specification involved in pronephros development	GO:0039017	> 100	0.01
	pronephric field specification	GO:0039003	> 100	0.01
	negative regulation of mesenchymal cell apoptotic process involved in metanephric nephron morphogenesis	GO:0072305	> 100	0.01
1	metanephric glomerular capillary formation	GO:0072277	80.16	3.36E-02
	metanephric glomerulus vasculature morphogenesis	GO:0072276	80.16	3.35E-02
	metanephric glomerulus morphogenesis	GO:0072275	80.16	3.34E-02
2	regulation of cellular response to X-ray	GO:2000683	> 100	5.35E-03
	endodermal digestive tract morphogenesis	GO:0061031	> 100	7.99E-03
	positive regulation of ureteric bud formation	GO:0072107	> 100	1.14E-02
3	pattern specification involved in pronephros development	GO:0039017	99.73	1.80E-02
	pronephric field specification	GO:0039003	99.73	1.80E-02
	negative regulation of mesenchymal cell apoptotic process involved in metanephric nephron morphogenesis	GO:0072305	99.73	1.80E-02
4	positive regulation of mesenchymal cell apoptotic process	GO:2001055	96.97	3.43E-02
	negative regulation of neuromuscular junction development	GO:1904397	96.97	3.41E-02
	corticospinal tract morphogenesis	GO:0021957	48.48	6.53E-03
5	regulation of presynaptic membrane organization	GO:1901629	> 100	2.85E-02
	negative regulation of mesenchymal cell apoptotic process involved in nephron morphogenesis	GO:0072040	85.31	2.71E-03
	regulation of mesenchymal cell apoptotic process involved in nephron morphogenesis	GO:0072039	85.31	2.69E-03
6	neuropilin signaling pathway	GO:0038189	32.61	1.48E-02
	glomerular capillary formation	GO:0072104	26.09	2.17E-02
	glomerulus vasculature morphogenesis	GO:0072103	26.09	2.16E-02

E

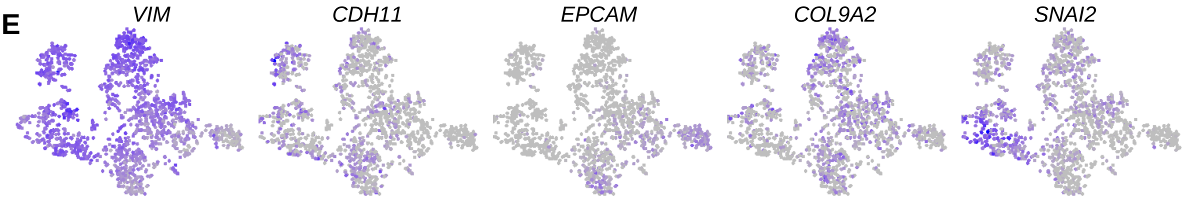

Development • Supplementary information

Fig. S2: (A) t-SNE plot coloured by cell cycle stages in the different clusters. **(B)** Violin plots showing the number of UMIs, expressed genes and percentage mitochondrial genes in each cluster. **(C)** Number of cells in individual clusters. **(D)** Associated GO biological process terms for the significantly up-regulated genes within different clusters. **(E)** t-SNE plots for additional selected marker genes related to nephrons.

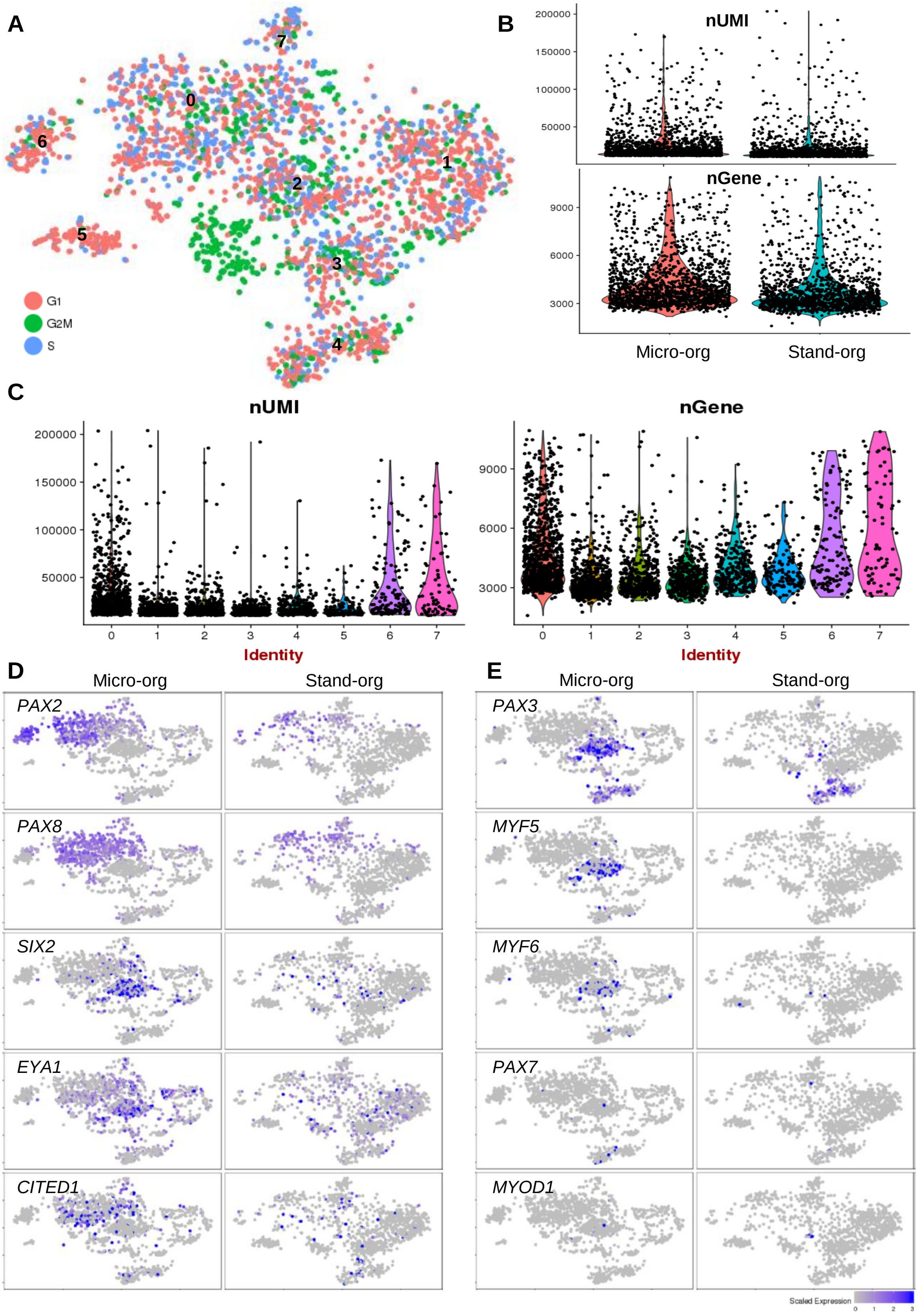
A
0
1
2
3
4
5
6
7
G1
G2M
S
B
nUMI
200000
150000
100000
50000
nGene
9000
6000
3000
Micro-org
Stand-org
C
nUMI
200000
150000
100000
50000
0 1 2 3 4 5 6 7
Identity
nGene
9000
6000
3000
0 1 2 3 4 5 6 7
Identity
D
Micro-org
Stand-org
PAX2
PAX8
SIX2
EYA1
CITED1
E
Micro-org
Stand-org
PAX3
MYF5
MYF6
PAX7
MYOD1
Scaled Expression
0 1 2 3

Fig. S3: (A) t-SNE plot coloured by cell cycle stages in the integrated analysis of micro- and standard organoid scRNA-Seq data. **(B)** Violin plots for the number of UMIs and expressed genes in the standard and micro-organoid data. **(C)** Number of UMIs and genes within clusters. **(D and E)** t-SNE feature plots for nephron marker genes in standard and micro-organoid scRNA-Seq data.

Fig. S4

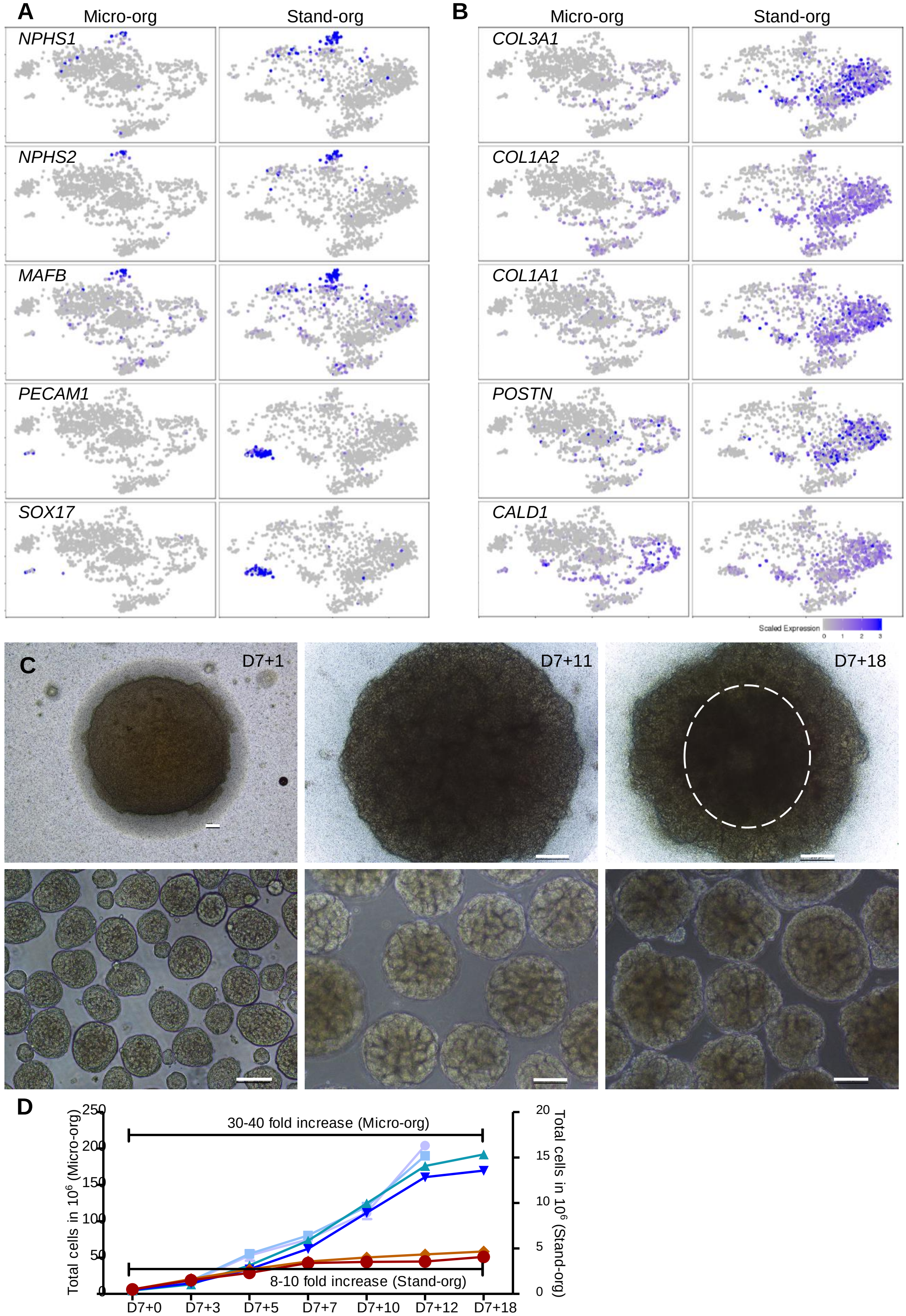

Development • Supplementary information

Fig. S4: (A and B) t-SNE plots for cell type marker genes in standard and micro-organoid. *NPHS1, NPHS2* and *MAFB* mark podocytes, *PECAM1* and *SOX17* mark endothelial cells, *COL3A1, COL1A2, POSTN, COL1A1, LGALS1,* and *GALD1* mark stroma. **(C)** Bright field images of organoid development across time course among standard organoid (upper panels) and micro-organoids (lower panels) (scale 200 μm). **(D)** Total number of organoid-derived kidney cells over time within kidney micro-organoids and standard kidney organoids.

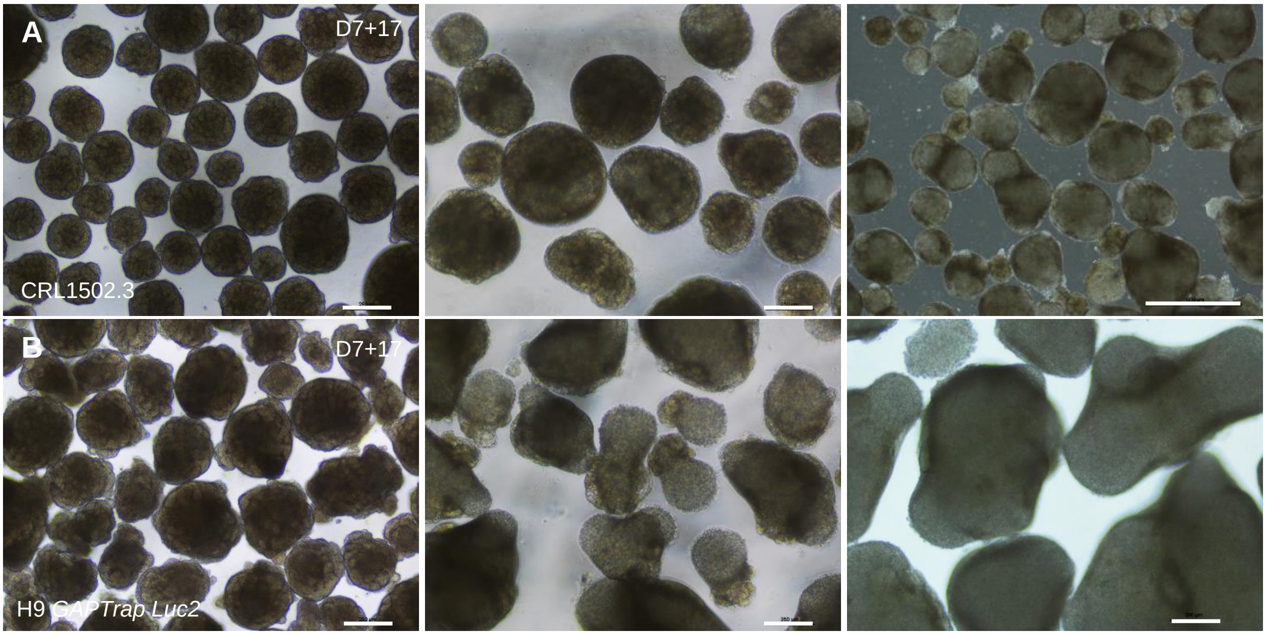

Fig. S5: (A and B) Bright-field images of extended culture of kidney micro-organoids in suspension using CRL1502.3 and H9 *GAPDH Luc2* cell lines (scale 250 μm),

Table S1

Click here to Download Table S1

Table S2. Human gene specific primers used in this study.

	Forward	Reverse
GAPDH	AGCCACATCGCTCAGACAC	GCCCAATACGACCAAATCC
PAX2	AGGACCAGTTTCCATAGACTGC	GGGAGGGGGCATCAAGTA
WT1	TGTCAGCGAAAGTTCTCCG	GCTGAAGGGCTTTTCACCTG
LHX1	ATGCAACCTGACCGAGAAGT	CAGGTCGCTAGGGGAGATG
OSR1	ATAGAACCTGTGGGTCACAAGGAC	GGGACAATGTTGGAGAGGTGG
SIX1	AAAGGGAAGGAGAACAAGGATAG	GGAGCCTACATGATTACTGGG
EYA1	ATCTAACCAGCCCGCATAGC	GTGCCATTGGGAGTCATGGA
WNT11	TGAACGACTCGGAACTCGTC	CGCTTCCGTTGGATGTCTTG
GATA3	GCCCCTCATTAAGCCCAAG	TTGTGGTGGTCTGACAGTTCG
C-RET	CCGCACACGGCTGCATGAGA	AAGGTGCCTGGGGGTCGGTT
FOXD1	GACTGTGAGTTCATCAGCGGC	TGACGAAGCAGCGTTGAGCGA
NPHS1	GTCTGCACTGTCGATGCCAATC	CCAGTTGGCATGGTGAATCCG
NPHS2	CTGTGAGTGGCTTCTTGTCCTC	CCTTTGGCTCTTCCAGGAAGCA
SYNPO	TAGGGTGTTGGGCTGGATGTCA	AGGAGGTGAGATGCAGCACACT
CUBN	AACTTCCTAATCCCCAGCGG	GTCCACCTCCTCAGTTCCTG
HNF4A	ACCCTCGTCGACATGGACA	GCCTTCTGATGGGGACGTG
CD31	AAACCACTGCAGAGTACCAG	GCCTCTTTCTCCAGTGTTGT
LAMA5	AACCAGATGAGCATCACATTCCTG	ACAGTGTTGCGCGTCTCCGTAT
CDH6	AGATGCTGCAAGGAATCCTGTC	CCATAGCAGTGTTTCTCGGTCAA
CDH1	CACCCTGGCTTTGACGCCGA	AAACGGAGGCCTGATGGGGCG

Development • Supplementary information
